# Perceptions and acceptability of the controlled human malaria infection (CHMI) model in The Gambia: a qualitative study

**DOI:** 10.1038/s41598-023-35752-x

**Published:** 2023-05-29

**Authors:** Edgard Diniba Dabira, Alexandra Fehr, Nathalie Beloum, Jean-Pierre Van geertruyden, Jane Achan, Annette Erhart, Melisa Martinez-Alvarez, Umberto D’Alessandro

**Affiliations:** 1 Medical Research Council Unit The Gambia at London School of Hygiene and Tropical Medicine (MRCG at LSHTM), Fajara, The Gambia; 2 Global Health Institute, Faculty of Medicine and Health Sciences, University of Antwerp, Wilrijk, Belgium; 3 London School of Hygiene and Tropical Medicine, London, UK; 4 Malaria Consortium, London, UK

**Keywords:** Malaria, Health policy, Medical ethics, Public health

## Abstract

Controlled human malaria infection (CHMI) studies, *i.e.* the deliberate infection of healthy volunteers with malaria parasites to study immune response and/or test drug or vaccine efficacy, are increasingly being conducted in malaria endemic countries, including in sub-Saharan Africa. However, there have been few studies on the perceptions and acceptability of CHMI by the local communities. This qualitative study assessed the perception and acceptability of such studies in The Gambia following the first CHMI study conducted in the country in March–May 2018. Data were collected through non-participant observation, in-depth interviews and focus group discussions and analyzed using NVivo 12 software with an inductive-deductive approach. Sixty-seven participants were involved, including volunteers enrolled in the CHMI, community stakeholders and members of the Gambian Ethics Committee. Respondents expressed a positive view about CHMI. Key motivating factors for participation were the financial compensation, comprehensive health checks, and willingness to support malaria research. Risks associated with participation were considered low. Concerns raised included the frequency of bleeding and the blood volume collected.

## Introduction

Progress towards the vision of a malaria-free world has recently stalled, with 2 of the 4 goals of the Global technical strategy (GTS) for malaria off track^1,2^. Indeed, the expected reduction by at least 40% of both malaria mortality and morbidity by 2020 as compared to the 2015 levels has not been achieved. To reverse this trend, novel and innovative interventions are needed. These may include safe and effective vaccines and drug products with the potential of interrupting malaria transmission to ultimately achieve elimination^3,4^. However, vaccines and drug products development is a lengthy, complex process requiring substantial resources and time^5,6^. For example, the timeframe for vaccine development can be up to 18 years, with a cost between USD200 million to USD900 million and an overall probability of success of approximately 11%^5,7^.

Controlled human malaria infection (CHMI) studies consist of deliberate infection of healthy volunteers with malaria parasites, either by mosquito bites or direct injection of sporozoites or parasitized erythrocytes. These well-controlled proof of concept studies allow to both understand the development of the immune response against malaria infection, and to rapidly screen for potential vaccine and drug candidates^8–12^. They are substantially smaller (only tens of participants), shorter (can be completed in a few weeks), and less expensive than large clinical trials, and allow for the selection of candidate vaccines and drug products worthy of further investigation in larger field trials^5,13^. Unlike large field trials, the CHMI enables the investigators to entirely control the exposure to malaria parasites both in terms of strain and dose^6,14^. Thus, CHMI studies are valuable tools to accelerate vaccines and drugs products development.

Since the first well-documented CHMI with laboratory-reared infectious mosquitoes carried out in 1986 at the US Walter Reed Army Institute of Research (WRAIR), the number of CHMI studies have increased in the United States and Europe, and are increasingly being conducted in malaria-endemic countries, including in sub-Sahara Africa^6,15,16^. Because of population differences related to naturally acquired immunity, genetics, nutrition, etc., it is important to conduct CHMI studies in malaria-endemic countries rather than ‘northern’ malaria-naïve countries to allow early assessment of vaccine and drug efficacy in a population with pre-existing malaria immunity^17,18^. Conducting CHMI studies is also important in building the capacity and infrastructures of research institutions in endemic countries and enables African researchers to become involved in the earlier stages of vaccine or drug development^9,10^. However, the deliberate infection of healthy volunteers with malaria parasites violates the fundamental principle in medicine of “*primum non nocere”* (“first, do not harm”) and raises multiple ethical concerns. In CHMI studies, volunteers have little or no direct benefit from their participation. Instead, CHMI studies aim at advancing scientific knowledge for public health gains. Selecting a specific category of volunteers from the same area raises the concern that relatively privileged populations may be the primary beneficiaries of research conducted in underprivileged populations. Time commitments, discomfort of being infected, and study procedures can be burdensome. In addition, volunteers are often confined (which can prevent them engaging in their daily activities) for close monitoring and to prevent inadvertent transmission to a third-party^14^. Financial payment for compensating time lost may lead to undue inducement, especially in populations of low socio-economic status^19^. Therefore, careful, and rigorous ethical reviews are vital to ensure the safety of volunteers while maximizing scientific gains. In addition to ethical concerns, it is crucial to understand the perception and acceptability of CHMI studies in communities where they are done as this is key for their success^20^. However, until now, few qualitative studies have evaluated perceptions and acceptability of volunteers and communities’ stakeholders. As an ancillary study of the first CHMI study conducted in The Gambia in 2018, we assessed perceptions and acceptability of a CHMI study among volunteers’ and malaria endemic rural communities.

### Controlled human malaria infection study in The Gambia

The CHMI study was implemented between March and May 2018 at the Clinical Services Department of the Medical Research Council Unit The Gambia at the London School of Hygiene and Tropical Medicine (MRCG at LSHTM), situated in Kanifing, an urban area near the capital city Banjul^21^. Its aim was to assess the parasite kinetics and functional immunity in Gambian adults following PfSPZ Challenge administration. Briefly, this was an open-label, non-randomized clinical trial. The study screened healthy male volunteers aged 18–35 years from tertiary learning institutions; and a total of 19 individuals were enrolled in the study. All participants were administered an intravenous dose of 3.2 × 10^3^PfSPZ Challenge and were closely followed for 28 days. As financial compensation for the time lost by participating in the study, each volunteer received USD 160 over the period of follow-up.

## Methods

### Study settings

The qualitative study was implemented in two very different settings. The first was Kanifing, West Coast Region, where the volunteers for the CHMI study were recruited. Kanifing is an urban environment and part of the greater Banjul (capital city) area. The second setting was Basse, Upper River Region, where most malaria research projects are conducted. It is a rural setting, approximately 375 km from Banjul, with moderate but highly seasonal malaria transmission, mainly between September and December^22,23^.

### Study design and sampling strategy

The study used a qualitative method to gain insight of CHMI study volunteers experiences while in the study and community stakeholders perception and acceptability of such studies. The study was conducted between January and December 2019. Convenient sampling was used to select respondents enrolled in the CHMI study and the Ethics Committee members. None of the Ethics Committee members was involved in the process of granting approval of the CHMI study. Respondents were contacted through phone calls and the purpose of the study was explained to them. Those who agreed to participate were invited for a face-to-face interview. A purposive sampling was performed to select individuals from the community from both study areas to include household heads, adults male and female and religious leaders. The same strategy was used to select adults, male and female in the community for the focus group discussions (FGD). The respondents were selected to ensure maximum variation in personal viewpoints, age, sex, education and professional background, and to reflect differences in residence (urban *versus* rural setting).

### Data collection

In-depth interviews (IDI) and focus group discussions (FGDs) were used to collect data from respondents identified through communities within MRCG at LSHTM network. IDIs were conducted with CHMI study participants, Ethics Committee members and adult individuals in the communities. Interviews lasted 20–40 min and were conducted face to face and audio-recorded. The respondents chose a convenient time and location for the interviews. An interview guide with open-ended questions was used to facilitate reflection and dialogue with the respondents. FGDs were used to collect data from adult in the communities. The size of each FGD varied between 6 and 8 members and included a total of 20 males and 16 females, and each FGD lasted between 40 and 60 min. Data collection guides, developed based on the ethical issues arising from literature, were designed to capture perceptions and acceptability of the CHMI model in The Gambia. Both IDIs and FGDs were conducted in English and local languages, depending on the language preference of the participants.

### Data analysis

All interviews were transcribed, translated into English where indicated (interviews conducted in local languages) and managed using NVivo 12 software. Data were analyzed using thematic analysis with an iterative process. We used both deductive and inductive approaches for this qualitative study. The deductive part consisted of developing a set of questions based of the ethical issues arising from literature. The questions were grouped into six main domains including benefits, perceived risks, reasons for participation, selection of volunteers, decision-making process, and financial compensation. Within these six domains of inquiry, we used a general inductive approach and generated codes from the raw data. We created codes based on key words and then grouped them on the basis of their similarities into categories. To ensure the quality of coding, we adopted a double blind procedure whereby the two members of the research team (EDD and NB) generated codes and themes independently. Then they met and went through the material and the codes in order to refine and finalize both the codes and themes and proceed for the description and interpretation. The initial sample analysis was set at 15 and the themes were compared to subsequent interviews to identify emerging new themes so as to decide whether saturation was reached^24^.

### Ethics clearance

The study was reviewed and approved by the Gambia Government MRC joint Ethics Committee (SCC 1615). Written informed consent was sought from all participants for the interviews (IDIs and FGDs) and for audio recording. All methods were carried out in accordance with relevant guidelines and regulations.

## Findings

### Participants characteristics

A total of 67 participants were recruited; 43 males and 24 females whose age ranged from 18 and 70 years. We conducted a total of 31 IDIs (n = 31) and 6 FGDs (n = 36). In Kanifing, IDIs included 8 respondents enrolled in the CHMI study, 3 members of the EC and 8 respondents from the community while the two FGDs included 12 respondents. In Basse, IDIs were conducted with 12 respondents while the 4 FGDs included 24 respondents. Respondents were farmers, religious leaders (Imam), head of households, students and self-employed (Table 1).

**Table 1:** Characteristics of the respondents in the CHMI qualitative study.

Characteristics	Volunteers enrolled in the CHMI study	Gambia Ethics Committee members	Community stakeholders	Community stakeholders
	n = 8	n = 3	n = 20	n = 36
	In-depth interviews		Focus group discussions	
*Age range*				
18–35	8	–	5	20
36–45	–	–	5	10
46–65	–	3	5	6
65–70	–	–	5	–
*Gender*				
Male	8	3	12	20
Female	–	–	8	16
*Education level*				
None	–	–	4	6
Primary education	–	–	6	10
Secondary education	–	–	8	16
Tertiary education	8	3	2	4
*Occupation*				
None	–	–	–	6
Student	8	–	5	8
Subsistence farmer	–	–	8	12
Religious leader(Iman)	–	–	2	2
Self–employed/business	–	1	3	4
Employed/Civil Servant	–	2	2	4
*Location*				
Kanifing (West Coast Region)	8	3	8	12
Basse (Upper River Region)	–	–	12	24
	Total in-depth interviews		Total focus group discussions	
Kanifing (West Coast Region)	19			2
Basse (Upper River Region)	12			4

### Perceived benefits

Overall, the eight respondents previously enrolled in the CHMI study considered their participation as a positive experience. There were several points that were considered extremely positive, including the detailed information sheet provided prior to enrolment and the opportunity to ask questions for clarification; the extensive health check, including laboratory tests, that confirmed they were healthy prior to enrolment; the accommodation in a residence near the study clinic throughout the follow up period, with free access to internet; the professionalism of the research staff; and the financial compensation. Being accommodated together with other study participants was particularly appreciated for the opportunity to meet other study participants with whom they could establish friendly links as stated by one of the respondents *“I really enjoyed the CHMI study, I made new friends and had access to internet all throughout, that was great for me as a student to learn more through internet… The research team was so nice and friendly. I also made some money which definitely helps me a lot because as a student I have no income….”* (CHMI study participant 1).

Respondents were also happy to contribute improving knowledge on malaria by participating in this research project and appreciated that the CHMI study was conducted in The Gambia where malaria is a major public health problem. A student stated: *“There is no malaria in the developed world, CHMI study should be conducted where malaria is a problem like in The Gambia, so it is important and useful for us to volunteer and help scientists to know more about malaria and how to treat it”* (CHMI study participant 2).

Similarly, respondents from the EC commented on the impact of CHMI studies, arguing of the public health benefits. They felt that the ethical acceptability of the CHMI studies should be related to generating scientific knowledge that is particularly relevant to the local communities. They argued that if scientifically sound, a CHMI study should be conducted in countries like The Gambia where malaria is a public health problem. As stated by a member of EC “*Yes…! It makes sense to conduct such studies in The Gambia. Our people suffer a lot from malaria”* (EC member 1). Although they acknowledged the expertise and experience of MRCG at LSHTM in malaria research, they stated that the capacities of other stakeholders involved in granting permission for the conduct of the CHMI study, such as National Regulatory Authority and the EC, should be strengthened by specific trainings. Such trainings should build scientific, ethical review, and regulatory capacity for CHMI studies in The Gambia to ensure that these studies are conducted according to the highest standards as mentioned by an EC member: *“Although these studies have been conducted in the western world for a long time, they [CHMI studies] are new in The Gambia. As members of Ethics committee we need to understand the implications of such studies through specific trainings so that we can ensure that the studies are conducted to high standard for the safety of the study participants”* (EC member 2)*.*

Most respondents from the wider community, regardless of provenance and socio-economic status, indicated that it would be acceptable to conduct CHMI studies in The Gambia though the majority had never heard of such studies as indicated by a respondent *“I have never heard such studies conducted within our community, I know that mosquito bites cause malaria but to infect a healthy person with malaria, this is my first time of hearing such….”*(Male, community stakeholder 1, Basse). However, most respondents argued that malaria is highly prevalent and such studies should be welcomed as they would improve scientific knowledge which would ultimately help defeat malaria in The Gambia. However, these views were influenced by the technical expertise and trust in the long-standing collaboration between MRCG at LSHTM and the local communities as mentioned by a stakeholder in Basse *“MRC has been working in The Gambia for a long time, they [MRCG at LSHTM] help a lot our communities. I remembered I beneficiated from MRCG at LSHTM treatment, and I know they do a good job, so I feel like supporting MRCG at LSHTM work which in return would contribute to advance malaria research and this would be beneficial to our communities in the future.* (Male, community stakeholder 2, Basse).

### Perceived risks

All respondents enrolled in the CHMI studies indicated that they had experienced minor malaria symptoms during the follow-up period but without consequence on their daily activities. Most interviewees from the wider community and the members of EC thought the risk of participating in the CHMI study was low. The perceived risks in relation to safety were mitigated by several factors, including previous exposure to malaria, malaria as a curable disease, and trust in the Research Institution (MRCG at LSHTM) as indicated by a student *“I had malaria several times in my life, and I know if you get malaria there are good medicines even when you have severe malaria doctors can cure you…. So, for me I did not see any problem or a major risk of taking part of this study conducted by MRCG at LSHTM”* (CHMI study participant 3). On the other hand, a few respondents expressed some concerns about the frequency and volume of blood collections. A student said: *“You know…. we do not have enough blood…. and you want to collect blood again and again.… how would that person feel at the end?”* (CHMI study participant 4). Additionally, a few respondents raised concerns about the study schedule which had interfered with their own university lectures schedule. They admitted that the information was provided in the information sheet. However, they did not anticipate these potential disruptions as mentioned by a student “*At the start, I did not realize it would be difficult to concurrently attending classes and the study schedule, I missed few classes especially during the first week post challenge where we had frequent medical checks…. I was in the study clinic in the morning and in the afternoon and we were also asked to stay for observation …. So, I ended up missing classes …”* (CHMI study participant 5).

#### Motivation for participation and participation in future studies

Financial payment as a compensation for time lost was the first motivating factor when interviewing the respondents enrolled in the CHMI study. They mentioned that the money was used for their daily expenses, including school fees, restaurant bills, mobile phone credit cards, data for internet, books, and stationery. A student stated: *“The compensation was great, it helped me a lot… I paid part of my school fees and used some of the money to buy personal stuffs and for transport fare”* (CHMI study participant 6). Similarly, respondents from the wider community valued the monetary compensation. However, there were mixed answers when asked what a fair compensation would be. While elderly respondents felt that they should rely on what the research institution offers*,* young respondents regardless of the provenance expressed that the amount should be higher than what was given to the volunteers in the CHMI study. Nonetheless, respondents from the EC stated that the amount of financial payment to the study participants as compensation for their time lost was fair given the number of days volunteers were confined in a residence and the discomfort and burden of the study procedures. However, one respondent from the EC stated that the amount provided is higher than what a student would expect if involved in a professional activity and raised a concern for potential inducement. He mentioned that the amount of cash should be indexed on a stipend for a student if this category was to be recruited “*I felt the financial compensation is quite high given the Gambian context. A good approach could be to align the compensation with local wage or students’ stipend”* (EC member 3).

While respondents from the EC viewed the free comprehensive medical check as intrinsic to the CHMI study, most respondents enrolled in the CHMI study considered it as the second motivating factor for participation. They stated that a comprehensive medical review would not have been possible otherwise as commented by a student *“For a long time, I wanted to go to hospital for check up, I wanted a doctor to check my heart…. I know you guys are doing electrocardiogram (ECG) tests…., in town this exam is quite expensive, and I cannot afford it…. You know, we students we have no money to check our health condition.* (CHMI study participant 7).

Another motivating factor was the willingness to contribute to malaria research. Irrespective of provenance, age, education and professional background, respondents felt their participation was important as a way to contribute to malaria research which would ultimately benefit their community. Nonetheless, the expertise, trust, and longstanding relationship of MRCG with the local communities influenced their willingness to participate.

When asked if they will be happy to participate in future CHMI studies, most respondents from the CHMI study and community stakeholders reported their willingness to participate in future CHMI studies. *“I did not encounter any problems while participating in the study, I know there is no problem in participating…. So, I would take part in another CHMI study”* (CHMI study participant 2). Some mentioned that they will recommend their friends and family to join the study as mentioned by a stakeholder in the community “ *I will encourage my family members to participate in future CHMI studies*” (Male, stakeholder 3, Kanifing).

#### Decision making process

All respondents enrolled in the CHMI study consulted their family before taking the decision to participate. Opinions of the family members were key in the decision-making process. The decision- making process involves informing parents and respected community members, discussing and balancing inconvenience and advantage, and obtaining permission or favorable opinion from the family members. While most young respondents stated they will first inform and discuss with their mother to obtain their permission, some elderly respondents argued that they will seek advice from religious leaders (Imam) and the elderly in the community. Parents’ opinions and approval was key and was considered as a norm and societal value. Respondents felt it is important to receive a “blessing” from their parents before taking part to a study. A student said: *“That is how it should be as a human being you need to have parents or guardians who look after you so whatever you want to engage yourself into inform them and seek for advice and blessings”* (Male, Community stakeholder 4, Basse).

#### Selection of participants

Respondents from the wider community indicated that the selection of participants should include all community members, regardless of the level of education. Most respondents from Basse, where most MRCG at LSHTM malaria projects are implemented, stated that community members can understand what the study is about provided the right information is given in local languages as stated by a community stakeholder: *“Many people in our communities are illiterate…. but when they are involved in MRCG at LSHTM work, they understand… they sign (thumbprint) the informed consent, they follow the study procedures… and I never heard that a participant has been excluded because he failed to understand the study”* (Community stakeholder 5, Basse). Some added that visual aids (videos and pictorials) could facilitate comprehension of this new concept.

## Discussion

This study reports on the perceptions and acceptability among participants and community stakeholders of the first CHMI study implemented in The Gambia. Overall, most respondents expressed a positive view about this type of study, with all prior CHMI volunteers showing enthusiasm on their participation. Similar positive experiences were also reported by Njue et al. in Kenya^25^ . The financial compensation offered in exchange of the time lost was a key motivation as volunteers were asked to stay at a hotel near the clinical services for safety reasons and to facilitate their follow up. The agreement of the volunteers’ parents for their participation was extremely important as well as the long term and well-established expertise of the MRCG at LSHTM and its longstanding relationship with the local communities. Given the nature of the CHMI, namely the infection of healthy volunteers with malaria, its implementation would have been extremely difficult, if not impossible, without the trust of the local communities have in the implementing institution.

Despite these positive perceptions and attitudes, some concerns for study procedures were expressed and were mainly related to both the frequency and volume of blood sampling. This is not surprising as similar concerns are usually expressed for studies with a less intense blood sampling schedule^26^. In Kenya, CHMI volunteers were also concerned about blood sampling which was found to be burdensome^25^. Collecting blood samples from individuals recruited into clinical research projects in sub-Saharan Africa can be challenging and often related to rumours of “blood stealing” or “blood selling”^26,27^. Such rumours represent a social diagnosis and a logical attempt to make sense of the clinical trial in today’s world that should be countered by communicating with research participants in culturally appropriate ways and by addressing their concerns. CHMI volunteers received exhaustive explanations about the study and its procedures and none of them expressed any fear about the improper use of the blood samples collected. Nevertheless, they probably underestimated the inconvenience related to the daily visits, blood draws and confinement, as happened also in Kenya and in the USA^25,28^.

Because CHMI studies are complex and logistically challenging, it is generally thought that recruiting study participants with higher level of education would facilitate the informed consent process as these participants would be in better position to understand the study aim and procedures than uneducated ones^14,29^. This is the reason why most CHMI studies included volunteers with at least a tertiary education level^9,10^. Nevertheless, many stakeholders in our qualitative study expressed strong support for the recruitment of volunteers with a lower education level or even illiterate as they would be able to comprehend study procedures if explained in local languages and with visual aids. A qualitative study carried out in Kenya reported that less educated individuals are able to provide adequate informed consent, especially with well-designed community engagement and multiple opportunities to discuss and clarify the study procedures^25^. Moreover, selecting only individuals of a certain level of education may be unethical and would raise several concerns. Indeed, highly educated individuals do not fully represent the communities to be targeted by an intervention such as a new vaccine. Less educated individuals are probably at higher risk for malaria and excluding them would be unfair, resulting in their exclusion from the benefit of study. Therefore, fairness in the selection of CHMI volunteers is key as well as evaluating the specific vulnerabilities of the potential study population as it would reduce the risks of burden and harms^16^.

There has been considerable debate in the scientific community regarding monetary payment of study participants involved in clinical research^30^. It has been argued that the perceived risk and level of burden of CHMI justified higher compensation for study participants^20,31^. However, this may unduly induce study participation by impairing decision-making, with participants potentially accepting more risks than they would usually accept and thus “invalidating” the informed consent process ^14^. In our study, the financial payment was a key motivating factor for accepting to be part of the CHMI, similar to Njue et al. in Kenya and Kraft et al. in the USA^25,28^. However, many volunteers acknowledged that the comprehensive medical checks, trust in the research institution were important determinants of their participations..

Because monetary payment had positive effects on respondents’ willingness to participate in research^20,30^, it is vital that the Institutional Review Board (IRB) and Ethics Committees cautiously determine the appropriateness of the level of financial payment. A study in Kenya where volunteers received USD250-500^10,25^ resulted in a short-lived controversy in the local media^32,33^. Indeed, an article in a local newspaper titled “Want Cash? Volunteer for a dose of malaria parasite” suggested this was a quick and easy economic activity for participants. In response to the article, the research Institution issued a statement detailing the rationale of the study, its procedures, and the reason for the level of payment. In The Gambia, most members of the Ethics Committees indicated the level of compensation was fair after extensive discussions before the study received ethical approval^34^. However, young respondents in the wider community suggested CHMI volunteers should be compensated with a larger amount given the level of burden, raising the question on how to determine an acceptable amount. Adequate payment remains a subject of debate in which it is difficult to reach a consensus among the various stakeholders^34,35^. Dickert and Grady recommend the adoption of the wage payment model by which payment is based solely on standard wage payment for unskilled labour, with additional payments being made for uncomfortable procedures^36^. This model reduces undue inducement concerns, standardizes payment schedules, and establishes a system in which payment is based on the contribution subjects make, consistent with the principle of equal pay for equal work^34^. Lynch and colleagues suggest payment of the volunteers either in the form of compensation (for time and research-related burden and inconvenience), reimbursement (reimbursement of out-of-pocket expenses) or incentive (as a mean to encourage recruitment and retention)^37^. Although different views were expressed regarding payment, our findings indicate that Ethics committee members favor a combination of compensation and reimbursement methods.

Findings of our study indicate that CHMI studies are acceptable for Gambian communities. Similar results were found in Kenya which may suggest that CHMI studies would be acceptable by communities in other part of sub-Saharan Africa, the region with the highest burden of malaria^10,25,38^. However, despite acceptance of the challenges studies, community engagement may differ from one country to another. Community engagement should be determined locally based on an appropriate program and mutual trust between communities and research institutions. With willingness of communities to take of CHMI studies, conducting such studies in malaria endemic areas offer several benefits including building and reinforcing local capacity in term of scientific expertise, clinical facilities, laboratory diagnostic, governance and regulatory; and offer the opportunities to accelerate or streamlining development of vaccine and treatment relevant for sub-Saharan Africa^8^.

## Limitations

The method of selection of the respondents using a professional network of MRCG at LSHTM’s fieldworkers may have resulted in selection bias. However, to minimize it, we ensured that respondents varied in terms of age, education and professional background, and provenance. Another bias is that this qualitative study was an ancillary study of the CHMI trial and data collection was done by MRCG at LSHTM’s staff involved in both studies. This may have resulted in respondent bias as some respondents may not have felt at ease in discussing or revealing some negative views about the study. A further limitation is the reporting bias whereby only relevant themes are reported but readers are aware that reporting bias is inherent to such studies. Nevertheless, the study provides an insight into the perception and acceptability of the CHMI model in The Gambia.

## Conclusion

There have been recent calls for more CHMI studies in malaria endemic settings to accelerate vaccine development and test new interventions in communities with the highest disease burden^8,16^. The impact of CHMI studies on the communities justifies their conduct in malaria endemic countries such as The Gambia. Weighing the potential benefits and burdens associated with CHMI studies requires a careful and rigorous ethical and scientific review of the study protocol and should also consider local communities’ perception and acceptability. Findings from our study indicate that CHMI studies are acceptable for Gambian communities but are greatly influenced by the longstanding trust and relationship between local communities and MRCG at LSHTM. Nonetheless, all stakeholders involved in CHMI studies (investigators, IRB, EC, and local communities) need to adopt policies and guidelines to adapt CHMI studies to the local context and ensure risks are appropriately minimized during their implementation.

## Data Availability

Data are available from the corresponding author on a reasonable request.
